# Epidemiological Trends and Burden of Adult Kidney Cancer in Saudi Arabia: A 42‐Year Retrospective Analysis (1982–2023)

**DOI:** 10.1002/cnr2.70603

**Published:** 2026-06-12

**Authors:** Arwa F. Flemban, Saeed M. Kabrah, Hanaa Alahmadi, Lama S. Alahdali, Lama S. Maksood, May A. Alqurashi, Omnia J. Kutbi, Hanan M. Abd Elmoneim, Abdulaziz Bakhsh, Turky Almouhissen, Samar Nabeel Ekram, Halah T. Albar, Waseem Tayeb

**Affiliations:** ^1^ Department of Pathology, Faculty of Medicine Umm Al‐Qura University Makkah Saudi Arabia; ^2^ Department of Clinical Laboratory Sciences, Faculty of Applied Medical Sciences Umm Al‐Qura University Makkah Kingdom of Saudi Arabia; ^3^ Faculty of Medicine Umm Al‐Qura University Makkah Saudi Arabia; ^4^ Department of Pathology, Faculty of Medicine Minia University Minia Egypt; ^5^ General and Specialized Surgery Department, College of Medicine Taibah University Madinah Saudi Arabia; ^6^ College of Medicine University of Jeddah Jeddah Saudi Arabia; ^7^ Department of Genetics, College of Medicine Umm Al‐Qura University Makkah Saudi Arabia; ^8^ Department of Physiology, Faculty of Medicine Umm Al‐Qura University Makkah Saudi Arabia; ^9^ Department of Surgery, Division of Urology King Abdullah Medical City at Holy Capital Makkah Saudi Arabia; ^10^ Clinical Sciences Department Fakeeh College for Medical Sciences Jeddah Saudi Arabia

**Keywords:** epidemiology, kidney cancer, renal cell carcinoma, Saudi Arabia

## Abstract

**Background:**

Cancer epidemiology differs between males and females due to variation in exposure to risk factors. Kidney cancer incidence and mortality have increased globally, yet long‐term sex‐specific trends in Saudi Arabia remain insufficiently characterised. Kidney cancer incidence in Saudi Arabia has increased steadily over time, with an average annual percentage change (APC) of 3.22% over the study period.

**Aim:**

This study aimed to analyse long‐term trends in kidney cancer burden in Saudi Arabia and to compare incidence, mortality, years of life lost (YLLs) and disability‐adjusted life years (DALYs) by sex over the period 1982–2023.

**Methods:**

A retrospective observational study was conducted using non‐overlapping data from the Saudi Cancer Registry and the Global Burden of Disease 2021 study. Adults aged 20 years and older diagnosed with kidney cancer between 1982 and 2023 were included. Age‐standardised incidence, mortality, YLLs and DALYs were analysed by sex. Temporal trends were assessed using log‐linear regression models and APC was calculated.

**Results:**

A cumulative total of 34 247 adult kidney cancer cases was identified between 1982 and 2023. Over the 42‐year period, incidence increased by 3.22% annually. The increase was faster in females than in males (3.65% vs. 2.99%, *p* < 0.001). YLLs rose by 1.45% per year and DALYs by 1.73%, both showing greater increases among females. Despite this, the overall burden remained consistently higher in males across all indicators. Disease burden increased progressively with age and was highest in older adult populations.

**Conclusion:**

Kidney cancer burden has increased substantially in Saudi Arabia over the past four decades. Although males experience a higher overall burden, females show a faster rate of increase. These findings highlight the need for sex‐specific prevention strategies, improved early detection, and strengthened national surveillance. Addressing modifiable risk factors such as smoking, obesity and hypertension remains a priority for public health intervention.

## 1 Introduction

Globally, the incidence and mortality of kidney cancer are increasing annually [1], and the disease ranks among the most common malignancies in developed countries [2], including countries in Europe [3], North America [3, 4], Australia and Japan [2]. The burden of kidney cancer extends beyond clinical outcomes, with substantial economic impact reported in earlier analyses; although contemporary estimates are required to reflect current healthcare costs [5]. In 2020, kidney cancer accounted for 431 288 new cases and 179 368 deaths worldwide, reflecting a substantial and increasing global burden. Incidence has risen from approximately 160 000 cases in 1990 to nearly 390 000 cases in 2021, as demonstrated by recent global burden analyses [2, 6]. The availability of accurate and up‐to‐date cancer statistics is essential to guiding national health strategies, including resource allocation, prioritisation of high‐risk populations, development of preventive programmes and implementation of early detection strategies.

Several studies have evaluated trends in kidney cancer globally and across specific regions [2, 6]. Despite these efforts, country‐specific epidemiological data remain limited in many regions [2, 4, 5, 6]. In Saudi Arabia, previous studies on kidney cancer have primarily focused on risk factors [7, 8, 9] and clinicopathological characteristics, rather than on population‐level epidemiological trends [7, 8, 9]. Hypertension, smoking and diabetes mellitus have been consistently identified as major risk factors in the Saudi population [8, 9, 10, 11]. Global evidence has also identified additional risk factors linked to kidney cancer, including elevated body mass index [12, 13, 14], occupational exposure to carcinogens such as trichloroethylene [15], dietary patterns [16, 17], chronic kidney diseases [18, 19], kidney stones [20, 21] and physical inactivity [22]. However, the contribution of these risk factors has not been adequately quantified in Saudi populations due to the lack of large‐scale, nationally representative cohort data.

Recent studies have explored the application of artificial intelligence and machine learning techniques for kidney cancer detection and classification using imaging data, particularly computed tomography (CT) [23, 24, 25, 26]. These approaches have demonstrated promising accuracy in tumour classification and diagnostic support. However, such studies are primarily focused on image‐based diagnosis and model performance and do not address population‐level epidemiological trends, disease burden or long‐term temporal changes. Consequently, while advances in artificial intelligence contribute to clinical decision‐making, there remains a lack of comprehensive national epidemiological analyses that integrate incidence, mortality and burden metrics over extended time periods [25, 26]. Addressing this gap is essential for informing public health strategies and healthcare planning.

The epidemiology of kidney cancer in Saudi Arabia remains insufficiently characterised. Existing Saudi studies are mostly limited to single‐centre and regional analyses [7, 8, 10, 11, 27, 28, 29, 30]. These studies described clinicopathological features, risk factor profiles and treatment outcomes and have consistently reported a male predominance, clear cell renal carcinoma as the common subtype and a diagnosis typically occurring in middle‐aged and older adults [7, 8, 10, 11, 27, 28, 29, 30]. Although these studies provide valuable insights, they do not capture national trends over time. To date, no study has examined nationwide kidney cancer trends in Saudi Arabia over an extended period using comprehensive epidemiological indicators. Such analysis is essential for understanding the evolving disease burden and informing healthcare planning and policy decisions.

Data from the Saudi national cancer registry indicate a rising incidence of kidney cancer, with a reported increase of approximately 33% and a tendency towards late‐stage presentation [10, 30, 31]. This trend is concerning given the absence of structured early detection programmes in the country. Currently, most cases of adult kidney cancer in Saudi Arabia are diagnosed incidentally in asymptomatic patients during routine imaging examinations for unrelated conditions [7, 8, 10]. Surgical management remains the primary treatment modality, with increasing adoption of minimally invasive techniques that improve patient outcomes and reduce morbidity [7, 10, 11, 27, 30].

Despite the growing burden of kidney cancer in Saudi Arabia, its national epidemiological profile remains inadequately defined. Existing Saudi studies are largely limited to single‐centre or regional cohorts and do not provide a long‐term national assessment of incidence, mortality and overall disease burden. This creates an important evidence gap that limits the ability to monitor temporal trends, identify sex‐specific differences and guide prevention, early detection and health policy at the national level. Accordingly, this study aimed to provide a nationwide long‐term analysis of adult kidney cancer in Saudi Arabia from 1982 to 2023 using multiple epidemiological indicators, including incidence, mortality, years of life lost (YLLs) and disability‐adjusted life years (DALYs), with sex‐specific comparisons. By using nationwide data over four decades, this study offers a more comprehensive assessment of disease burden and supports evidence‐based planning for prevention, early detection and healthcare resource allocation in Saudi Arabia.

The main contributions of this study are summarised as follows. First, it provides a nationwide long‐term analysis of adult kidney cancer burden in Saudi Arabia over a 42‐year period. Second, it integrates non‐overlapping data from the Saudi Cancer Registry and Global Burden of Disease 2021 estimates. Third, it evaluates multiple epidemiological indicators, including incidence, mortality, YLLs and DALYs. Fourth, it identifies sex‐specific differences in temporal trends. Finally, it provides evidence to support national cancer control strategies.

The remainder of this paper is organised as follows. The Methods section describes the study design, data sources and statistical analysis. The Section 3 presents the epidemiological trends in incidence, mortality, YLLs and DALYs. The Discussion interprets the findings in the context of existing literature and public health implications. Finally, the Conclusion summarises the key findings and outlines directions for future research and policy development.

## 2 Method

### 2.1 Study Design

A retrospective observational study was conducted to examine the epidemiological trends and burden of kidney cancer in Saudi Arabia over a 42‐year period (1982–2023). This longitudinal design was selected to enable systematic analysis of temporal patterns in disease incidence, mortality and health‐related burden at the national level using pre‐existing large‐scale secondary datasets. The study adhered to the Strengthening the Reporting of Observational Studies in Epidemiology (STROBE) guidelines.

### 2.2 Data Sources

Data were obtained from two primary sources: the Global Burden of Disease 2021 database and the Saudi Cancer Registry (SCR). The Global Health Data Exchange platform was used to access and extract GBD estimates. GBD 2021, maintained by the Institute for Health Metrics and Evaluation, provided standardised modelled estimates of kidney cancer burden for Saudi Arabia from 1990 to 2021. For 1982–1989 and 2022–2023, data were obtained exclusively from the Saudi Cancer Registry. To avoid double‐counting, the two sources were used only in non‐overlapping periods: GBD estimates for 1990–2021 and SCR counts for 1982–1989 and 2022–2023. No record‐level merging was performed.

The SCR is Saudi Arabia's national population‐based cancer registry, established formally in 1994 under the Ministry of Health. It covers all administrative regions of the Kingdom and operates through a passive and active case‐ascertainment system drawing upon multiple data sources, including hospital discharge records, pathology laboratory reports, death certificates and outpatient clinical documentation. Data are collected by regional cancer registration offices and submitted to the central SCR for quality control and consolidation. The registry applies internationally standardised criteria for case definition and coding, using the International Classification of Diseases for Oncology (ICD‐O) and transitioning from ICD‐9 to ICD‐10 coding during the mid‐1990s. Although formal completeness studies for the SCR are limited in the published literature, the registry is widely used as the authoritative source for national cancer burden estimates in Saudi Arabia and has been employed in multiple international comparative analyses, including Global Burden of Disease modelling. As with many cancer registries in middle‐income settings, early‐period (pre‐1994) data should be interpreted with some caution, given that the registry's coverage and operational capacity were progressively developed following its formal establishment.

For the present analysis, kidney cancer data were first extracted separately from the Saudi Cancer Registry and the GBD 2021 database according to their respective temporal coverage. Adult cases aged 20 years and older were then identified and age‐standardised incidence, mortality, YLLs and DALYs were compiled by sex and age group. Temporal trends were then evaluated within predefined intervals using log‐linear regression and annual percentage change (ACP) was calculated for each indicator. This workflow enabled a consistent long‐term assessment of kidney cancer burden across the full study period.

To avoid double‐counting, the two sources were used only in non‐overlapping periods: GBD estimates for 1990–2021 and SCR counts for 1982–1989 and 2022–2023. No record‐level merging or duplication adjustment was performed. Both sources were analysed within a common age‐standardised framework to support comparability across time periods.

### 2.3 Study Population

The study population comprised adults aged 20 years and older diagnosed with kidney cancer in Saudi Arabia between 1982 and 2023. The cumulative total of adult kidney cancer cases across the full study period was 34 247. For age‐stratified analyses, the population was categorised into 16 five‐year groups (20–24 through ≥ 95 years), consistent with GBD standard procedures.

### 2.4 Outcome Measures

The primary outcomes were age‐standardised rates (per 100 000 population) of kidney cancer incidence, mortality, DALYs and YLLs, stratified by sex and age group. All age‐standardised rates were computed using the GBD World Standard Population, a demographically stable reference population that enables valid comparisons across time, sex and geographic subgroups. This standard population is consistent with the one used in all GBD 2021 publications and is documented in the GBD 2021 methods appendix (IHME 2022). DALYs were computed as the sum of YLLs and YLDs. YLLs were derived by multiplying the number of deaths at each age by the standard life expectancy at that age based on the GBD reference life table.

YLDs were estimated following the Global Burden of Disease 2021 methodology, using the formula: YLDs = Prevalence × Disability weight. The disability weight for kidney cancer applied in GBD 2021 analyses is 0.049 (95% UI: 0.031–0.072) for the diagnosis and primary treatment phase, representing moderate functional impairment associated with the disease and its treatment. This weight was derived through a structured expert consensus process involving pairwise comparisons of health states, as described in the GBD 2013 disability weights measurement study [32] and subsequently updated in GBD 2016 and GBD 2021 iterations. Disability weights applied in the GBD framework are documented in the GBD 2021 Results Tool [33] and are consistently applied across all national estimates to enable valid international comparisons. Prevalence estimates used in YLD calculations were drawn from the GBD 2021 modelled estimates for Saudi Arabia, which apply DisMod‐MR 2.1, a Bayesian meta‐regression tool, to synthesise available epidemiological data.

### 2.5 Temporal Data Consistency

Given the 42‐year study horizon, potential variability in diagnostic standards, registry completeness and coding practices across decades warrants explicit consideration. Three principal sources of temporal heterogeneity exist: (1) the transition from ICD‐9 to ICD‐10 coding in Saudi Arabia's health system during the mid‐1990s may have caused transient reclassification effects; (2) the SCR underwent progressive expansion and coverage improvement, particularly after the establishment of the National Cancer Registry in 1994, implying that pre‐1994 case ascertainment was likely less complete and (3) the widespread adoption of CT and MRI from the early 2000s onwards substantially increased incidental detection, which is a driver of apparent incidence trends independent of true disease burden. The GBD framework applies backcasting and smoothing algorithms to partially mitigate these discontinuities, but residual artefacts cannot be entirely excluded. The observed acceleration in all four indicators during 2002–2011 is likely partly attributable to improved diagnostic capacity rather than a pure increase in underlying incidence.

### 2.6 Handling of Early‐Period Data (1982–1989)

**FIGURE 1:**
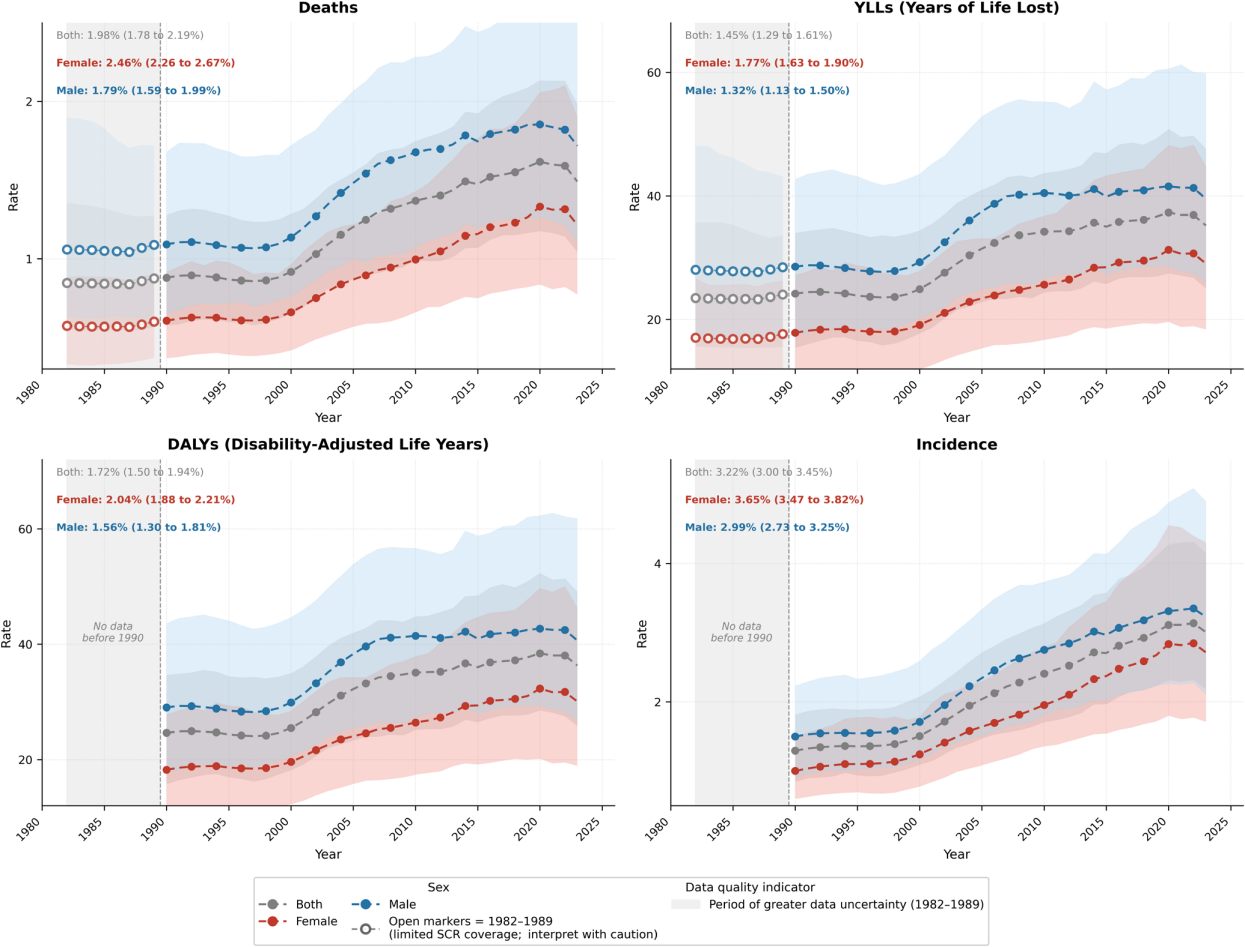
Trends in age‐standardised kidney cancer epidemiological indicators in Saudi Arabia by sex (1982–2023). This figure presents longitudinal trends in age‐standardised incidence, deaths, disability‐adjusted life years (DALYs) and years of life lost (YLLs) for kidney cancer in Saudi Arabia from 1982 to 2023, stratified by sex (male, female and both sexes combined). Annual data points from 1982 to 1989 (open markers and grey‐shaded background) are derived from early Saudi Cancer Registry (SCR) data and should be interpreted with caution because registry completeness and case ascertainment were less established during this period. Data from 1990 onwards (filled markers) represent the period of more complete national cancer registration and surveillance. Shaded bands surrounding each trend line represent 95% uncertainty intervals. Annual percentage change (APC) estimates with corresponding 95% confidence intervals are displayed within each panel. Across all epidemiological indicators, males consistently exhibited higher absolute rates throughout the study period, whereas females demonstrated steeper relative increases over time. The figure highlights the progressive increase in the burden of kidney cancer in Saudi Arabia over the past four decades while illustrating sex‐specific differences in temporal trends.

The graphical analysis in Figure 1 originally began in 1990, reflecting the limited availability of reliable annual data prior to that year due to incomplete SCR coverage. To address reviewer concerns regarding the discrepancy between the graphical display and the APC time frame, Figure 1 has been extended to include the 1982–1989 period. Annual data points from 1982 to 1989 are displayed as open circles with grey shading to indicate the period of greater data uncertainty. This presentation allows readers to observe the full time series for which APC estimates are reported, while explicitly signalling the reduced reliability of early‐period estimates. However, APC calculations for the 1982–1991 period incorporate available SCR aggregate data from 1982 onwards, the earliest year for which validated national counts were available. For this early interval, confidence intervals are correspondingly wider, reflecting greater uncertainty in the data. APC estimates for the 1982–1991 interval should therefore be interpreted with caution. The confidence intervals for this period are correspondingly wider than for subsequent intervals, reflecting genuine uncertainty in the underlying data. Readers are encouraged to focus primarily on sub‐period APCs for 1992 onwards, where data quality is more reliable, when drawing conclusions about long‐term epidemiological trends.

### 2.7 Statistical Analysis

All statistical analyses and visualisations were performed using Python (version 3.10; libraries: pandas, seaborn, statsmodels) and SPSS Statistics (version 28.0; IBM Corp., Armonk, NY).

Temporal trends in age‐standardised rates were analysed using period‐specific log‐linear regression models. This approach assumes that the rate of change within each sub‐period is approximately constant on a multiplicative (exponential) scale—an assumption consistent with the gradual accumulation of population‐level exposure to risk factors over time. To assess the robustness of this assumption, a single log‐linear model was also fitted to the full 1982–2023 time series; the consistency of period‐specific and overall APC estimates confirmed that no gross violation of the log‐linear assumption was present within individual sub‐periods. The study period was partitioned into four intervals: 1982–1991, 1992–2001, 2002–2011 and 2012–2023, each aligned with documented shifts in the Saudi healthcare system and cancer registration practices. Within each interval, the natural logarithm of the outcome rate was regressed on calendar year. APC was derived as APC = (*eβ* − 1) × 100. Separate models were fitted for each indicator and sex group. A summary APC for 1982–2023 was also calculated.

Between‐sex APC differences were assessed using *F*‐tests. Sex differences in age‐specific rates were explored using two‐sided independent‐samples *t*‐tests applied to age‐group‐specific rate estimates. Significance was defined at *p* < 0.05, *p* < 0.01 and *p* < 0.001.

### 2.8 Uncertainty Quantification

GBD 95% uncertainty intervals (UIs), generated via Monte Carlo simulations, were reported where available. Missing data were managed using GBD default imputation procedures [33]. These GBD imputation procedures use spatiotemporal Gaussian process regression and Bayesian meta‐regression to borrow strength from neighbouring years, related causes and comparable countries when direct data are sparse. In the Saudi Arabian context, this predominantly affects the pre‐1990 period and cause‐specific subcategories with small counts. The principal implication is that national‐level estimates may understate uncertainty for specific age‐sex subgroups in the early study period and this should be considered when interpreting granular subpopulation trends. A formal sensitivity analysis of imputation assumptions was beyond the scope of this study, but we acknowledge this as a limitation.

## 3 Results

### 3.1 Temporal Trends in Kidney Cancer Epidemiological Indicators in Saudi Arabia (1982–2023)

A cumulative total of 34 247 adult kidney cancer cases was estimated between 1982 and 2023, based on non‐overlapping Saudi Cancer Registry data and Global Burden of Disease modelled estimates. This study assessed the APC in kidney cancer incidence, mortality (deaths), DALYs and YLLs in Saudi Arabia over the 42‐year period from 1982 to 2023, stratified by sex and decade.

Incidence showed the highest overall APC across indicators. From 1982 to 2023, the average APC in incidence was 2.99% (95% CI: 2.68–3.30) among males and 3.65% (95% CI: 3.39–3.90) among females, with a combined APC of 3.22% (95% CI: 2.94–3.51). The difference in trend between sexes was statistically significant (*F* = 48.265, *p* < 0.001). Across sub‐periods, the highest APC occurred between 2002 and 2011 (male: 4.01%, female: 3.89%), while growth slowed between 2012 and 2023 (male: 1.44%, female: 2.68%) (Table 1).

**TABLE 1:** Annual percentage change in kidney cancer epidemiological indicators in Saudi Arabia (1982–2023).

	Measurement	Period	Male	Female	Both sexes	*F*‐statistic	*p*
0	DALYs	1982–1991	0.85% (0.85%–0.85%)	1.93% (1.93%–1.93%)	1.10% (1.10%–1.10%)	—	—
1	DALYs	1992–2001	0.50% (−0.28% to 1.27%)	0.67% (−0.05% to 1.39%)	0.45% (−0.31% to 1.22%)	0.265	0.614
2	DALYs	2002–2011	2.41% (1.57%–3.26%)	2.27% (1.90%–2.65%)	2.41% (1.71%–3.11%)	0.175	0.681
3	DALYs	2012–2023	0.16% (−0.14% to 0.47%)	1.14% (0.52%–1.77%)	0.54% (0.14%–0.95%)	12.583	**0.002**
4	DALYs	1982–2023	1.56% (1.28%–1.84%)	2.04% (1.82%–2.27%)	1.72% (1.46%–1.97%)	27.678	**0.000**
5	Deaths	1982–1991	0.50% (0.22%–0.78%)	0.88% (0.45%–1.31%)	0.60% (0.30%–0.90%)	5.979	**0.026**
6	Deaths	1992–2001	0.58% (−0.25% to 1.43%)	0.85% (−0.12% to 1.84%)	0.55% (−0.35% to 1.45%)	0.386	0.543
7	Deaths	2002–2011	3.19% (2.48%–3.91%)	3.28% (2.81%–3.75%)	3.28% (2.63%–3.93%)	0.109	0.745
8	Deaths	2012–2023	0.42% (−0.15% to 0.99%)	1.82% (0.97%–2.67%)	0.94% (0.29%–1.60%)	13.588	**0.001**
9	Deaths	1982–2023	1.79% (1.57%–2.01%)	2.46% (2.22%–2.71%)	1.98% (1.76%–2.21%)	90.747	**0.000**
10	Incidence	1982–1991	1.97% (1.97%–1.97%)	3.47% (3.47%–3.47%)	2.40% (2.40%–2.40%)	—	—
11	Incidence	1992–2001	1.54% (0.68%–2.40%)	2.07% (1.30%–2.84%)	1.64% (0.80%–2.48%)	2.441	0.138
12	Incidence	2002–2011	4.01% (3.28%–4.74%)	3.89% (3.58%–4.20%)	4.00% (3.42%–4.59%)	0.181	0.676
13	Incidence	2012–2023	1.44% (1.02%–1.85%)	2.68% (1.95%–3.41%)	1.93% (1.40%–2.46%)	16.880	**0.001**
14	Incidence	1982–2023	2.99% (2.68%–3.30%)	3.65% (3.39%–3.90%)	3.22% (2.94% to −3.51%)	48.265	**0.000**
15	YLLs	1982–1991	0.31% (0.10%–0.52%)	0.74% (0.33%–1.16%)	0.47% (0.22%–0.72%)	8.311	**0.011**
16	YLLs	1992–2001	0.46% (−0.31% to 1.24%)	0.62% (−0.09% to 1.34%)	0.42% (−0.35% to 1.18%)	0.204	0.657
17	YLLs	2002–2011	2.37% (1.53%–3.22%)	2.23% (1.85%–2.62%)	2.37% (1.67%–3.08%)	0.185	0.673
18	YLLs	2012–2023	0.13% (−0.17% to 0.44%)	1.11% (0.48%–1.73%)	0.51% (0.11%–0.92%)	13.913	**0.001**
19	YLLs	1982–2023	1.32% (1.14%–1.49%)	1.77% (1.60%–1.93%)	1.45% (1.29%–1.62%)	57.445	**0.000**

*Note:* Annual percentage change (APC) and corresponding 95% confidence intervals (CIs) in major epidemiological indicators of kidney cancer in Saudi Arabia between 1982 and 2023. The indicators include *Incidence*, *Deaths*, *Disability‐Adjusted Life Years (DALYs)* and *Years of Life Lost (YLLs)*. APC values represent the average annual rate of change during each period, calculated using a log‐linear regression model (log‐transformed rates regressed on calendar year). The analysis was stratified by sex (male, female and both sexes combined) and performed for successive intervals (1982–1991, 1992–2001, 2002–2011, 2012–2023) as well as the overall 1982–2023 period. The *F*‐statistic and *p*‐value correspond to between‐sex comparisons of APC slopes (testing equality of trends between males and females). Bold *p*‐values indicates statistically significant differences between male and females (*p* < 0.05).

Deaths from kidney cancer also rose over time. The overall APC in mortality was 1.79% (1.57–2.01) in males, 2.46% (2.22–2.71) in females and 1.98% (1.76–2.21) combined. Differences by sex were significant (*F* = 90.474, *p* < 0.001). The most rapid increase occurred between 2002 and 2011 (male: 3.19%, female: 3.28%). However, a slowdown in mortality growth was observed in 2012–2023, particularly among males (0.42%) (Table 1).

DALYs followed a similar pattern. The overall APC was 1.56% (1.28–1.84) for males and 2.04% (1.82–2.27) for females, with a combined rate of 1.72% (1.46–1.97). The sex difference in DALY trends was significant (*F* = 27.678, *p* < 0.001). The most rapid increases occurred between 2002 and 2011 (male: 2.41%, female: 2.27%), followed by a decline in the rate of increase in the last decade, especially among males (0.16%) (Table 1).

YLLs, representing premature mortality, also showed increasing trends. The overall APC was 1.32% (1.14–1.49) in males and 1.77% (1.60–1.93) in females, with a combined APC of 1.45% (1.29–1.62). Statistically significant differences between sexes were found (*F* = 57.445, *p* < 0.001). Between 2002 and 2011, the highest increases were observed (male: 2.37%, female: 2.23%), while growth again slowed in 2012–2023, most notably among males (0.13%) (Table 1).

Overall, all four indicators (incidence, deaths, DALYs and YLLs) exhibited increasing trends over the 42‐year period, with more pronounced increases observed among females in recent decades. While kidney cancer burden has risen in both sexes, statistical comparisons confirm that the pace of increase has been significantly faster in females, especially post‐2012.

### 3.2 Trends in Kidney Cancer Epidemiological Measures in Saudi Arabia (1982–2023)

Figure 1 illustrates the temporal trends in four major kidney cancer epidemiological indicators (incidence, deaths, DALYs and YLLs) in Saudi Arabia between 1982 and 2023, disaggregated by sex (male, female and both combined). These metrics are presented as age‐standardised rates per 100 000 population.

Incidence rates increased steadily for both, with a sharper rise observed from the early 2000s onwards. The average APC in incidence was highest among females (3.65%; 95% CI: 3.47–3.82), followed by both sexes combined (3.22%; 95% CI: 3.00–3.45) and males (2.99%; 95% CI: 2.73–3.25). Despite females showing the steepest APC, males consistently exhibited higher absolute incidence rates throughout the study period.

Mortality trends also rose across the decades. The age‐standardised death rate increased with an overall APC of 2.46% (95% CI: 2.26–2.67) in females, 1.98% (95% CI: 1.78–2.19) in both sexes and 1.79% (95% CI: 1.59–1.99) in males. Similar to incidence, mortality was persistently higher in males, although the rate of increase was more pronounced among females.

DALYs associated with kidney cancer showed a consistent upward trend across all sex groups. The highest APC was recorded in females (2.04%; 95% CI: 1.88–2.21), followed by both sexes (1.72%; 95% CI: 1.50–1.94) and males (1.56%; 95% CI: 1.30–1.81). This rise reflects the growing burden of disease in terms of both premature and non‐fatal health loss.

Figure 1 displays the complete study period (1982–2023). Data points from 1982 to 1989 are shown as open circles and should be interpreted with caution because registry coverage and completeness were less established during this period.

Overall, these trends demonstrate a significant and sustained increase in kidney cancer burden in Saudi Arabia over the past four decades. While males consistently bore a higher absolute burden, the faster rate of growth among females is noteworthy. These patterns underscore the need for intensified public health efforts, including prevention, early detection and equitable access to treatment, tailored to both sexes.

### 3.3 Kidney Cancer Epidemiological Measures by Age Group and Gender

**FIGURE 2:**
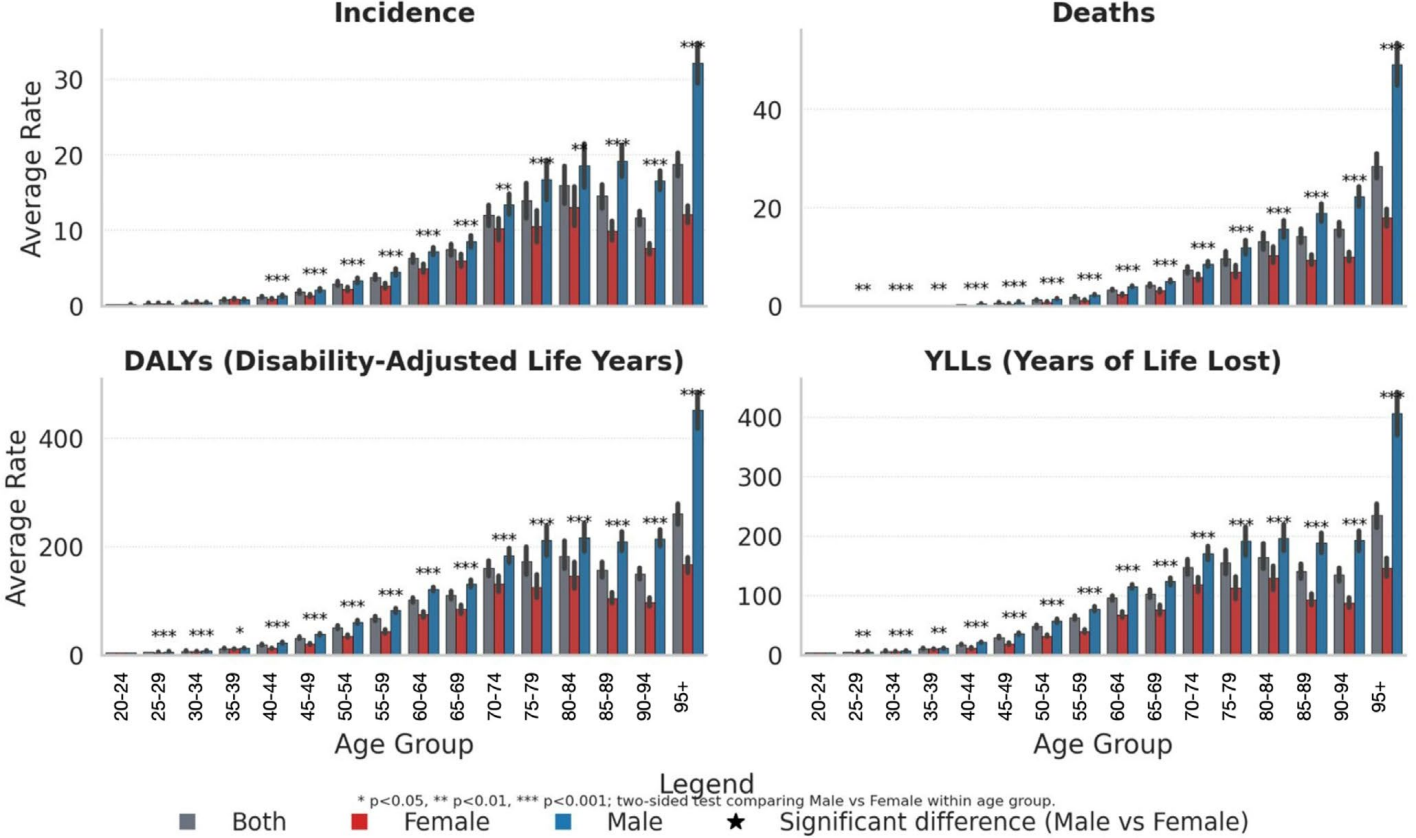
Average kidney cancer rates by age group and sex in Saudi Arabia (1982–2023) This figure presents four bar charts illustrating the average rates of key kidney cancer epidemiological measures—incidence, deaths, disability‐adjusted life years (DALYs) and years of life lost (YLLs)—across different age groups in Saudi Arabia from 1982 to 2023. Data are stratified by sex: male (blue), female (red) and both sexes combined (grey). Each bar represents the mean rate for a specific age group and asterisks indicate statistically significant differences between males and females within each group (*p < 0.05, **p < 0.01, ***p < 0.001), based on a two‐sided test. Rates increase with age across all indicators, with the highest values observed in older age groups.

Figure 2 presents the average rates of kidney cancer incidence, deaths, DALYs and YLLs stratified by age group and gender in Saudi Arabia between 1982 and 2023. The data show clear trends indicating increasing burden with advancing age, with notable differences between males and females across all metrics.

Incidence rates of kidney cancer were negligible in early childhood but began rising steadily from adolescence, peaking in the oldest age group (95+ years). Males consistently exhibited higher incidence rates than females across all age categories, with statistically significant differences emerging in early adulthood and persisting into old age. The incidence rate in males exceeded 30 per 100 000 in the oldest age group, compared to approximately 20 in females, highlighting a markedly higher burden in older men.

Mortality patterns, as shown in the deaths panel, mirrored those of incidence, with death rates remaining low in early age groups and rising sharply in middle and older adulthood. The highest death rates were observed in males aged 95 years and older, approaching 50 per 100 000, significantly higher than in females of the same age. The gap in mortality widened with age, underscoring the elevated fatality burden in elderly males.

DALYs, which reflect both premature mortality and morbidity, showed a pronounced increase with age, reaching their highest in the 95+ age group. While both males and females experienced increasing DALYs with age, males again bore a disproportionately higher burden, particularly in late adulthood. Across nearly all adult age groups, male DALY rates significantly exceeded those of females, with highly significant differences noted (*p* < 0.001 in most cases).

YLLs, a direct measure of premature death, also increased steeply with age and were substantially higher in males. From early adulthood onward, YLLs rose sharply, peaking in the oldest age group. Among males, YLLs exceeded 400 per 100 000 in the 95+ group, compared to about 250 in females. The gender gap in YLLs widened progressively with age, reflecting a heavier burden of premature death due to kidney cancer among elderly men.

Overall, the analysis demonstrates that kidney cancer burden escalates significantly with age, with males consistently showing higher incidence, mortality, DALYs and YLLs compared to females. These findings highlight the need for age‐ and gender‐sensitive strategies in kidney cancer prevention, early detection and management, especially targeting older adults and high‐risk male populations. Kidney cancer burden increases progressively with age across all indicators. Incidence, mortality, DALYs and YLLs rise steadily from early adulthood, reaching their highest levels in older age groups (≥ 75 years). Although the highest observed values appear in the oldest age categories, estimates in extreme age groups (≥ 95 years) should be interpreted with caution due to smaller population sizes and increased variability.

## 4 Discussion

Kidney cancer is globally recognised as a significant public health concern, ranking as the 14th most common cancer worldwide [1], with more than 431 000 new cases and 179 000 deaths reported in 2020 [1]. The burden of kidney cancer is notably higher in high‐income regions such as Europe, North America and Australia [2, 34, 35]. Renal cell carcinoma ranks among the Top 10 cancers in males in Saudi Arabia and represents a notable proportion of adult malignancies based on national and regional reports [5, 10, 36]. These findings should be interpreted in the context of the combined use of modelled GBD estimates and registry‐based SCR data, which together provide the most comprehensive available national picture but may introduce minor differences in estimation approaches across time periods.

The rising incidence and mortality rates in the Kingdom highlight the urgent need for evidence‐based strategies for prevention, early detection and effective management of kidney cancer. Updated epidemiological analysis from this study reveals a clear age‐related trend, with a sharp rise in incidence, deaths, DALYs and YLLs in individuals aged 50 and above and the highest rates observed in those aged 75–79 and older.

The findings of this study align with global trends, showing an increase in kidney cancer incidence over the past four decades [1, 5]. However, age‐related patterns were observed in Saudi Arabia [7, 8, 11, 27]. This differs modestly from global reports, in which the highest incidence is generally observed in older adults, often from 75 years onwards [1, 34]. In our data, incidence increased progressively with age, with a further increase in the ≥ 95 age group, which should be interpreted with caution because of greater variability in estimates at extreme ages. The rise observed from early adulthood, particularly, among males, may reflect earlier and sustained exposure to modifiable risk factors such as obesity, hypertension and smoking. Greater access to advanced imaging and more frequent incidental detection during routine or unrelated clinical evaluation may also contribute to earlier diagnosis. Together, these findings suggest age‐pattern differences between demographic structure, risk‐factor exposure and healthcare access.

The findings indicate that kidney cancer burden increases steadily with age, with the highest rates observed in older adult populations. This pattern is consistent across incidence, mortality, DALYs and YLLs. While the largest values are observed in the oldest age groups, estimates in extreme age categories should be interpreted cautiously due to potential variability. These findings align with established epidemiological patterns, where cumulative exposure to risk factors and age‐related biological changes contribute to increasing disease burden with advancing age. Previous studies in Saudi Arabia indicated that the incidence peaks at ages 50 and 55 [8, 11, 30, 37], whereas the highest age‐specific incidence rates in the present study were observed in older age groups, reflecting differences between age at diagnosis and age‐specific population burden. This pattern is consistent with age‐related accumulation of risk and reflects the influence of demographic structure, with a median population age of 31.8 years [38]. Although the overall burden is concentrated in older adults, the observable increase across middle‐aged groups highlights the role of early exposure to modifiable risk factors such as obesity, hypertension and smoking. These findings underscore the importance of preventive strategies targeting modifiable risk factors and support efforts to improve early detection through risk‐based approaches.

Kidney cancer incidence is increasing across the Gulf Cooperation Council (GCC) countries, with clear variation in magnitude between populations [5]. Recent regional analyses indicate that the burden is highest in the United Arab Emirates and Qatar, while Saudi Arabia and Kuwait also show a substantial and rising incidence. Across these countries, the age distribution follows a consistent pattern, with incidence increasing progressively with age and the highest burden observed in older adults, typically in the sixth to eighth decades of life [5]. A similar pattern was observed in our study, where incidence peaked in the 70–74 age category.

However, our findings also show a noticeable increase in burden across middle‐aged groups, which may be more pronounced than in some regional reports [5]. This difference may reflect earlier and sustained exposure to modifiable risk factors, as well as differences in population age structure and healthcare access. A consistent feature across GCC populations is male predominance, with higher incidence and mortality rates among males than among females [5]. Together, these findings suggest that while the overall age‐related pattern of kidney cancer in Saudi Arabia is broadly consistent with regional trends, there are differences in the distribution of cases across age groups that may be influenced by demographic and health system factors.

### 4.1 Risk Factors and Epidemiological Trends

National and subnational data indicate that a substantial proportion of Saudi adults are exposed to established modifiable risk factors for kidney cancer [8, 11, 30]. Obesity is highly prevalent in Saudi Arabia, with recent studies reporting rates ranging from approximately 20% to 39% among adults, with higher combined estimates when overweight is included, reflecting a substantial and persistent national burden [39, 40]. Hypertension is a major risk factor for kidney cancer. In Saudi Arabia, a recent systematic review and meta‐analysis estimated a pooled prevalence of 22.66% (95% CI: 18.95–26.60), with substantial variation across regions and populations [41]. Prevalence increases markedly with age, supporting its role as an important contributor to kidney cancer risk in the Saudi population. Smoking is a major modifiable risk factor for kidney cancer and remains common in Saudi Arabia. Recent Saudi data indicate tobacco use of approximately 19%–21% in the general population, with substantially higher prevalence among men. Early initiation is also common. In one recent Saudi study, 47% of smokers reported starting before 18 years of age [42, 43, 44]. This pattern is relevant to our findings, as the kidney cancer burden increased with age and remained consistently higher in males [43]. The higher prevalence of smoking among men, together with longer cumulative exposure, may partly explain the greater burden observed in males in this study. These findings support prevention strategies focused on weight control, smoking reduction and blood pressure management.

The faster annual increase observed among females may reflect a combination of behavioural, sociocultural and health‐system factors. In Saudi Arabia, obesity remains common among adult women [39] and high levels of physical inactivity have been reported [45], both of which are established contributors to kidney cancer risk. Emerging evidence also suggests increasing tobacco use among Saudi females, although overall prevalence remains lower than in males [46]. In addition, recent shifts in workforce participation among women may be associated with changes in lifestyle patterns, including reduced physical activity, increased occupational and environmental exposures and higher prevalence of comorbid conditions such as hypertension. Improved access to healthcare and diagnostic imaging may have further contributed to increased incidental detection over time. In high‐income settings with well‐established cancer registries, stage migration analyses have demonstrated that a substantial proportion of the observed increase in kidney cancer incidence is attributable to incidental detection of localised tumours during imaging for unrelated conditions, rather than to a true rise in disease burden [47]. Whether a comparable pattern has occurred in Saudi Arabia cannot be determined from the aggregate data available in this study. Nonetheless, the significant expansion of CT and MRI availability in Saudi Arabia, particularly in urban tertiary centres from the early 2000s onwards, is consistent with this mechanism and likely contributes to the acceleration in incidence APC observed in the 2002–2011 interval. Finally, the steeper APC observed in females may partly reflect a lower baseline burden at the start of the study period, whereby smaller absolute increases translate into larger relative changes. These findings suggest that sex differences in temporal trends are likely multifactorial and warrant further investigation using individual‐level data.

Diagnostic practices may also contribute to the observed patterns. The widespread use of cross‐sectional imaging, particularly CT and magnetic resonance imaging (MRI), has improved the detection and characterisation of renal masses [10, 48]. Many kidney tumours are now identified incidentally during imaging performed for unrelated conditions, often in asymptomatic patients [10]. This shift in diagnostic practice has been associated with increased detection of smaller and earlier‐stage tumours, which may influence the observed age distribution and contribute to apparent changes in incidence patterns. Improved access to advanced imaging in Saudi Arabia, particularly in urban centres, likely plays a role in this trend.

### 4.2 Molecular and Genomic Profiling of Renal Cell Carcinoma

The pathogenesis of renal cell carcinoma reflects an interplay between genetic susceptibility and environmental exposures. Recent genomic studies have refined the molecular landscape of RCC, identifying recurrent alterations in pathways related to hypoxia signalling, chromatin remodelling and cellular metabolism [49]. In clear cell RCC, inactivation of the VHL gene remains a central event, leading to dysregulation of hypoxia‐inducible factors and downstream angiogenic pathways. Additional recurrent alterations in genes involved in chromatin regulation, including PBRM1, SETD2 and BAP1, further contribute to tumour heterogeneity and progression [49].

Hereditary syndromes account for a minority of cases but provide important insight into disease biology. Germline mutations in genes such as VHL, FH, MET and FLCN are associated with distinct RCC subtypes and clinical behaviour [49]. Recent evidence also highlights the contribution of alterations in DNA repair pathways and broader genomic instability in a subset of tumours previously considered sporadic [50]. Advances in high‐throughput sequencing and integrative genomic analyses have enabled more precise molecular classification of RCC, with implications for prognosis and targeted therapy [51].

These developments are particularly relevant in the context of population‐based studies, as they provide a biological framework for understanding observed epidemiological patterns, including sex differences and variability in age distribution. However, region‐specific genomic data remain limited in Saudi Arabia, underscoring the need for future studies integrating molecular profiling with epidemiological surveillance.

Recent studies have begun to explore the genomic and molecular characteristics of renal cell carcinoma in Saudi Arabia, although available data remain limited. Early gene expression profiling studies in Saudi patients identified a range of differentially expressed genes and dysregulated pathways, suggesting potential population‐specific molecular signatures. More recent investigations have focused on histopathological and molecular correlations, confirming the predominance of clear cell carcinoma and highlighting variability in tumour biology across patients [52, 53]. Despite these advances, most studies are based on small cohorts and single‐centre data, limiting the generalisability.

The current evidence indicates that molecular profiling of RCC in Saudi Arabia is still in an early stage, with limited integration of large‐scale genomic sequencing or multi‐omics approaches. This gap restricts the ability to fully characterise tumour heterogeneity and its relationship to clinical outcomes in the local population. Expanding genomic research in Saudi Arabia is therefore essential to support precision oncology, improve risk stratification and better understand the biological mechanisms underlying the epidemiological patterns observed in this study.

### 4.3 Implications for Saudi Arabia

The findings of this study have direct implications for cancer control in Saudi Arabia. Kidney cancer incidence, mortality and burden have increased over time, with consistently higher rates in males and substantial burden in older adults. These trends support the need for stronger prevention and early detection strategies. At present, Saudi Arabia does not have a formal screening programme for kidney cancer. The expanding use of CT and MRI in routine clinical care has increased incidental detection, particularly, in urban centres and this may offer an opportunity for earlier diagnosis through structured risk‐based assessment in high‐risk individuals [9, 29, 30].

The increasing burden of kidney cancer in the Kingdom also reflects broader demographic and epidemiological changes, including population ageing and the high prevalence of obesity, hypertension and smoking [9, 29, 30]. These patterns indicate that prevention strategies should focus on modifiable risk factors as well as timely referral and diagnostic pathways.

Advances in molecular diagnostics and precision oncology may further improve kidney cancer care in Saudi Arabia. Radiogenomics, liquid biopsy, artificial intelligence and multi‐omics approaches are generating new opportunities for tumour classification, prognostic stratification and personalised treatment [10, 28, 29, 54]. Their implementation in the Saudi setting will require population‐specific validation and stronger integration of epidemiological, clinical, radiological and genomic data. National initiatives such as the Saudi Human Genome Project and the Saudi Cancer Registry provide an important foundation for this work.

### 4.4 Future Directions

The rising burden of kidney cancer in Saudi Arabia underscores the need for targeted research into disease determinants and prevention strategies. Future studies should examine the interplay between genetic susceptibility and environmental exposures in the local population, with particular attention to modifiable risk factors such as obesity, smoking and hypertension. Longitudinal, population‐based studies are needed to better define risk trajectories and identify high‐risk groups.

Advances in molecular epidemiology and multi‐omics approaches offer opportunities to deepen understanding of disease mechanisms and identify biomarkers for early detection and risk stratification. Integrating genomic, clinical and imaging data will be essential to support precision oncology approaches tailored to the Saudi population. In parallel, strengthening national cancer registries and linking them to biobanking and genomic initiatives will enable more robust epidemiological analyses and support evidence‐based health policy and planning.

### 4.5 Strengths and Limitations

This study provides a comprehensive, up‐to‐date epidemiological analysis of kidney cancer trends in Saudi Arabia over a 42‐year period. By combining Saudi Cancer Registry data with Global Burden of Disease estimates across non‐overlapping time periods, it offers one of the longest and most detailed assessments of kidney cancer burden currently available for the country. The incorporation of age‐standardised incidence, mortality, YLLs and DALYs allows a broad evaluation of disease burden beyond simple case counts. The use of APC estimates with confidence intervals also strengthens the interpretation of temporal trends and sex‐specific differences.

A key strength is the stratification of epidemiological indicators by age group, sex and time intervals. This approach provides a clearer picture of the demographic distribution of kidney cancer burden in Saudi Arabia and shows that males consistently experience higher incidence, mortality, DALYs and YLLs than females, despite the faster recent increase observed among females. The visual presentation of temporal and age‐specific patterns further improves interpretation and policy relevance.

The study relies on the Saudi Cancer Registry for national case counts and while this registry is the most comprehensive available data source for cancer epidemiology in Saudi Arabia, its completeness has not been formally evaluated using capture‐recapture or other independent methods. The pre‐1994 data in particular may underrepresent the true incidence burden, as registry coverage and infrastructure were consolidated progressively following the registry's formal establishment in 1994.

Several limitations should be acknowledged. First, the study is based on aggregate secondary data and therefore cannot evaluate individual‐level risk factors, treatment exposures, stage at diagnosis or survival outcomes. This limits causal inference and restricts the clinical interpretation of observed epidemiological trends. The study did not include stage‐at‐diagnosis, treatment or survival data, which limits the clinical interpretation of YLLs and DALYs. Future studies should integrate epidemiological and clinical registry data to evaluate survival trends and stage distribution in relation to long‐term burden estimates. The analysis was conducted at the national level and did not include regional stratification. Given the known geographic and healthcare access variability within Saudi Arabia, this may mask regional differences in kidney cancer burden. Future studies using disaggregated regional data are needed to better understand geographic variation and inform targeted public health strategies. Regional differences in risk factor distribution, healthcare access and diagnostic capacity may influence disease patterns across the country. Second, the analysis combines registry‐based counts and modelled GBD estimates across different time periods. Although these sources were used in non‐overlapping periods to avoid double counting, differences in estimation methods may introduce minor inconsistencies across decades.

Stage‐at‐diagnosis data were not available in a form suitable for systematic longitudinal analysis in this study. Although the Saudi Cancer Registry collects staging information, completeness of staging data, particularly, before 2000, is insufficient to support a reliable analysis of stage distribution trends across the full study period. Consequently, whether the observed increase in incidence partly reflects a shift towards earlier‐stage disease as a result of increased incidental detection, rather than a true increase in disease burden, cannot be definitively determined from the available data. This is an important limitation given the well‐documented role of widespread cross‐sectional imaging in driving stage migration for kidney cancer in other national settings. Future studies incorporating complete stage data should evaluate trends in early‐stage versus advanced‐stage presentation over time, which would substantially advance understanding of the contribution of diagnostic practice changes to the observed epidemiological trends in Saudi Arabia.

Third, the quality of long‐term trend estimation is affected by temporal variation in coding systems, registry completeness and diagnostic practice. Improvements in cancer registration, wider access to CT and MRI and increasing incidental detection may have contributed to apparent changes in incidence over time, particularly in the earlier years of the study period. APC estimates from the earliest interval should therefore be interpreted with caution. Fourth, the study does not include regional or socioeconomic stratification. As a result, intra‐national disparities in healthcare access, risk‐factor distribution and diagnostic capacity could not be assessed.

Finally, although the study discusses the likely role of modifiable and genetic risk factors, it does not quantify their direct contribution to kidney cancer burden in the Saudi population. Data on occupational exposures, environmental toxins, dietary patterns and population‐level genomic variation were not available for integration into the analysis. Future studies should link epidemiological surveillance with clinical registries, regional data and molecular profiling to provide a more complete understanding of kidney cancer in Saudi Arabia.

## 5 Conclusion

This study presents an updated and comprehensive epidemiological assessment of kidney cancer trends in Saudi Arabia spanning four decades (1982–2023). The results confirm a sustained and significant rise in incidence, mortality and disease burden, as measured by DALYs and YLLs, particularly among males. Notably, the burden has increased more steeply in recent decades among females as well, narrowing the gender gap in some indicators.

Kidney cancer burden increases with age, with the highest impact observed in older adult populations. Saudi Arabia has a relatively young population demographic profile. Earlier exposure to modifiable risk factors and improved access to diagnostic imaging technologies have increased incidental detection rates.

The gender disparity remains a critical theme, with males consistently exhibiting higher rates of incidence, mortality, DALYs and YLLs across nearly all age groups. However, the gap is less pronounced in recent years, particularly in burden indicators such as DALYs and YLLs, suggesting potential shifts in exposure to risk factors or healthcare‐seeking behaviour among females.

The growing prevalence of obesity, hypertension and smoking, all known risk factors for kidney cancer, continues to pose a major public health challenge. The rising trends observed in this study underscore the need for targeted prevention strategies, including lifestyle modification campaigns, tobacco control policies and blood pressure management. Public health interventions should focus not only on older populations but also on middle‐aged adults, given the earlier age of onset observed.

Despite improvements in detection, Saudi Arabia still lacks a formalised kidney cancer screening programme. The younger age at diagnosis highlights a critical opportunity to introduce opportunistic screening, particularly, during imaging for unrelated conditions, to catch early‐stage disease and improve prognosis. Public awareness campaigns tailored to high‐risk groups could further enhance early detection and reduce long‐term burden.

While this study offers a robust national picture of kidney cancer trends, it also reveals important gaps. The absence of regional‐level data, treatment outcomes and survival rates limits the ability to fully evaluate healthcare performance and disparities. Future research should integrate clinical, radiological and genomic data, especially as Saudi Arabia advances in cancer research infrastructure through initiatives like the Saudi Cancer Registry and Saudi Human Genome Project.

## 6 Main Points

1. Kidney cancer incidence in Saudi Arabia has increased markedly over the past four decades, with both males and females showing upward trends. From 1982 to 2023, the APC in incidence reached 2.99% for males and 3.65% for females, indicating a persistent rise across both sexes.
2. Mortality from kidney cancer has also grown significantly, with the APC in deaths between 1982 and 2023 reaching 1.79% in males and 2.46% in females. The burden of disease, as measured by DALYs and YLLs, also showed consistent increases, especially among males
3. Kidney cancer burden increases progressively with age, with the highest levels observed in older adult populations (≥ 75 years).
4. Modifiable risk factors such as obesity, smoking and hypertension are major contributors to the increasing kidney cancer burden. These factors are highly prevalent in the Saudi population, with early adulthood exposure compounding long‐term risk
5. There is an urgent need for targeted public health interventions, including improved screening, early detection programmes and risk factor reduction strategies. The findings also call for enhanced epidemiological research, particularly among younger adults and across different regions of Saudi Arabia, to inform future health policy and cancer control strategies.

## Author Contributions

**Arwa F. Flemban:** conceptualization, formal analysis, investigation, data curation, methodology, project administration, supervision, validation, visualization, writing – original draft, writing – review and editing. **Omnia J. Kutbi:** data curation, investigation, methodology, writing – review and editing. **Hanan M. Abd Elmoneim:** formal analysis, validation, visualization, writing – review and editing. **Halah T. Albar:** conceptualization, writing – review and editing, validation. **Abdulaziz Bakhsh:** visualization, investigation, writing – review and editing. **Saeed M. Kabrah:** conceptualization, data curation, formal analysis, investigation, methodology, validation, writing – review and editing, visualization. **Lama S. Maksood:** data curation, methodology, formal analysis, writing – review and editing. **Samar Nabeel Ekram:** data curation, methodology, writing – review and editing. **Turky Almouhissen:** formal analysis, visualization, writing – review and editing. **Hanaa Alahmadi:** formal analysis, methodology, validation. **May A. Alqurashi:** data curation, formal analysis, methodology, writing – review and editing. **Lama S. Alahdali:** data curation, methodology, writing – review and editing. **Waseem Tayeb:** conceptualization, data curation, validation, writing – review and editing.

## Funding

The authors have nothing to report.

## Ethics Statement

This study was reviewed and approved by the Committee for Research Ethics for the Health of the Makkah Region, Ministry of Health, Saudi Arabia (Approval No. H‐02‐K‐076‐0103‐268). Ethics review was required because the study utilised data from the Saudi Cancer Registry, a national health information system governed by the Ministry of Health; per national research governance regulations, formal IRB approval is a prerequisite for access to and use of Ministry of Health‐administered data, irrespective of study design or level of data identifiability. The study was conducted in accordance with the ethical principles of the Declaration of Helsinki and its subsequent amendments.

## Conflicts of Interest

The authors declare no conflicts of interest.

## Data Availability

The data that support the findings of this study are available from the corresponding author upon reasonable request.
